# Preliminary inventory and classification of indigenous afromontane forests on the Blyde River Canyon Nature Reserve, Mpumalanga, South Africa

**DOI:** 10.1186/1472-6785-4-9

**Published:** 2004-08-02

**Authors:** Mervyn C Lötter, Hans T Beck

**Affiliations:** 1 Terrestrial Services, Scientific Services, Mpumalanga Parks Board, Private Bag X1088, Lydenburg, 1120, South Africa; 2 Department of Biological Sciences, Northern Illinois University, DeKalb, IL 60115 USA

## Abstract

**Background:**

Mixed evergreen forests form the smallest, most widely distributed and fragmented biome in southern Africa. Within South Africa, 44% of this vegetation type has been transformed. Afromontane forest only covers 0.56 % of South Africa, yet it contains 5.35% of South Africa's plant species. Prior to this investigation of the indigenous forests on the Blyde River Canyon Nature Reserve (BRCNR), very little was known about the size, floristic composition and conservation status of the forest biome conserved within the reserve. We report here an inventory of the forest size, fragmentation, species composition and the basic floristic communities along environmental gradients.

**Results:**

A total of 2111 ha of forest occurs on Blyde River Canyon Nature Reserve. The forest is fragmented, with a total of 60 forest patches recorded, varying from 0.21 ha to 567 ha in size. On average, patch size was 23 ha. Two forest communities – high altitude moist afromontane forest and low altitude dry afromontane forest – are identified. Sub-communities are recognized based on canopy development and slope, respectively. An altitudinal gradient accounts for most of the variation within the forest communities.

**Conclusion:**

BRCNR has a fragmented network of small forest patches that together make up 7.3% of the reserve's surface area. These forest patches host a variety of forest-dependent trees, including some species considered rare, insufficiently known, or listed under the Red Data List of South African Plants. The fragmented nature of the relatively small forest patches accentuates the need for careful fire management and stringent alien plant control.

## Background

Mixed evergreen forests form the smallest, most widely distributed and fragmented biome in southern Africa [1]. Originally classified as Undifferentiated Afromontane forest [2], 44% of this vegetation type within South Africa has been transformed [2]. The forest biome only covers 0.56 % of South Africa [3], yet it contains 5.35% of South Africa's plant species [4]. These forests have a relatively high species richness of 0.58 species km^-2^, exceeding the grassland biome with 0.25 species km^-2 ^and lagging only the fynbos with 1.36 species km^-2^[5]. The forest biome occurring in Mpumalanga is now recognized as Mpumalanga Mistbelt Forest [6], and it covers only 0.51% of the province's surface area [7]. Cooper [8] conducted a study on the conservation status of indigenous forests within Transvaal, Natal and the Orange Free State, in which he estimated the Blyde River Canyon Nature Reserve's (BRCNR) forests at 352 ha. Recently, ownership of a 700 ha forest tract bordering on BRCNR at the base of the Drakensberg Escarpment was transferred to the Mpumalanga Parks Board (MPB). Therefore, it was thought that just over 1000 ha of forest occurred on BRCNR.

To begin to understand the nature and distribution of forests in the BRCNR landscape, we need to have an accurate surface area inventory, a species composition list, and an identification and classification of the communities. Standard ecological fieldwork coupled with remotely sensed data and GIS allow for an efficient analysis. Prior to this investigation of the indigenous forests on BRCNR very little was known about the size and floristic composition of the forest biome conserved within the reserve. In comparison, the grassland biome has received much attention on BRCNR with a monitoring program set up to assess the status of the grasslands. We report here an inventory of forest size, fragmentation, species composition and the basic floristic communities along environmental gradients.

### Study area

**Figure 1:**
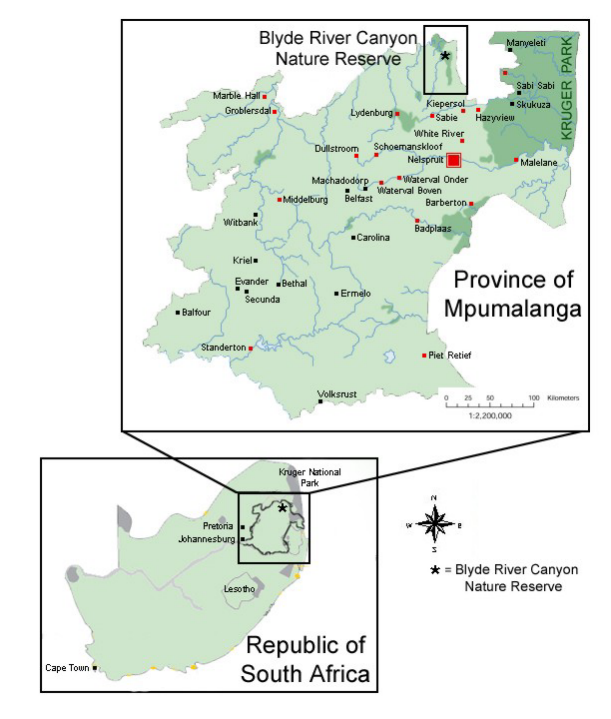
**Reserve location. **The Blyde River Canyon Nature Reserve is located in northeastern Mpumalanga Province, South Africa.

BRCNR is situated on the northern Drakensberg Escarpment, Mpumalanga Province, South Africa (24°40'S longitude, 30°51'E latitude; Figure 1). The Mpumalanga Parks Board administers this reserve, which is approximately 29 000 ha in size. The elevation on BRCNR ranges from 560 m to 1944 m above sea level. The stratigraphy of the northern Drakensberg Escarpment region is composed of sediment rock types (quartzite, shale, and dolomite) of the Transvaal Supergroup [9], dominated by Black Reef Quartzite and Wolkberg Group [10]. Rainfall varies from 541 mm to 2776 mm per annum. Variation in altitude and rainfall, associated with a landscape of geological and pedological extremes, has created a very diverse flora. The landscape is prone to lightning-induced burning [11] and is topographically complex, hosting a variety of habitats [12], including grassland plateaus, wetlands and sponge areas, grassland slopes, afromontane forest, riparian forest, moist woodlands, dry woodlands and shrublands. The vegetation type is classified as the Northeastern Drakensburg High-Mountain Sourveld ecoregion [13], and the reserve encompasses four veld types: Afromontane Forest, North-eastern Mountain Sour Grassland, Sour Lowveld Bushveld, and Mixed Lowveld Bushveld [14].

## Results

### Spatial distribution of forests

**Figure 2:**
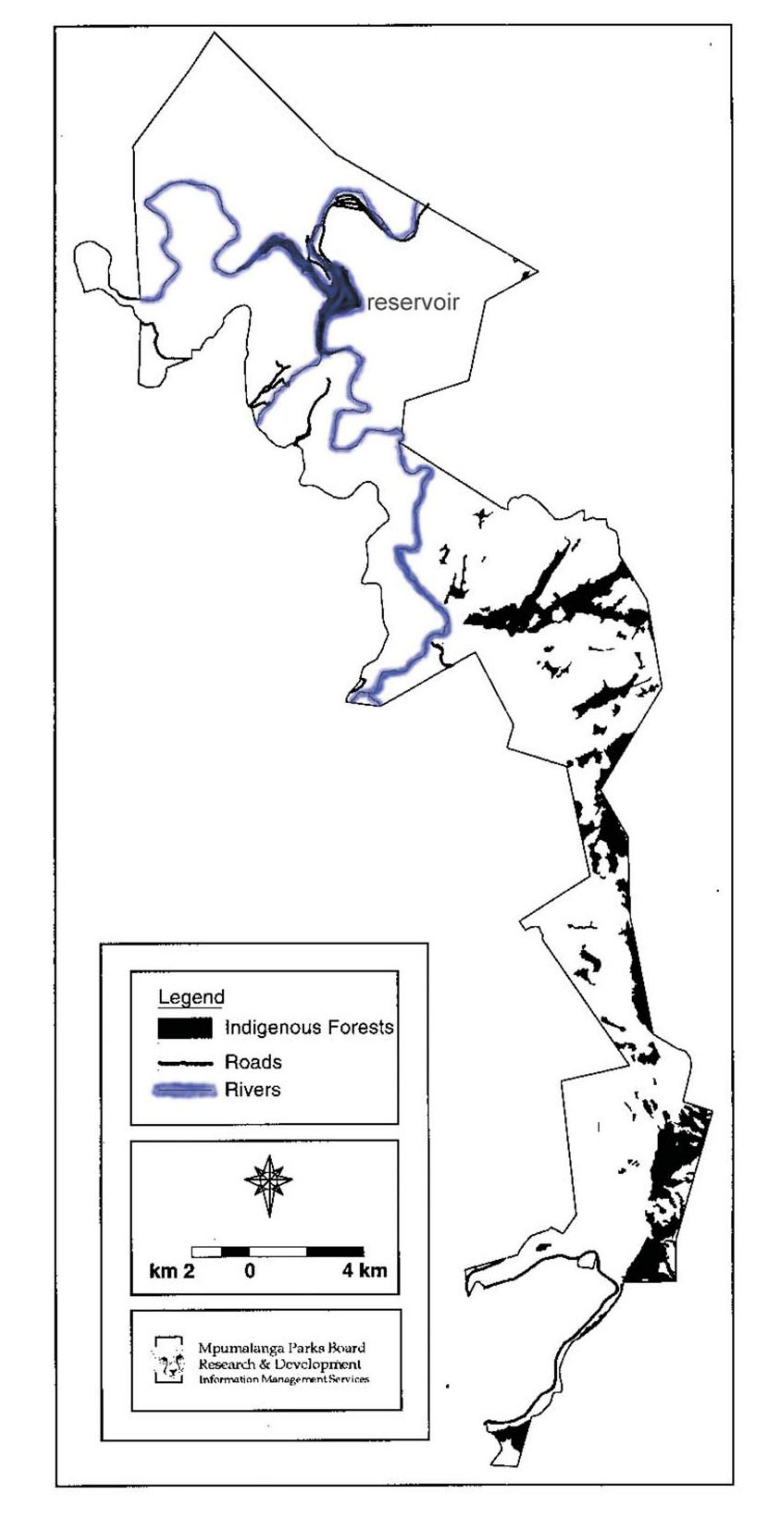
**Forest distribution. **Distribution of afromontane forest fragments in the Blyde River Canyon Nature Reserve.

**Figure 3:**
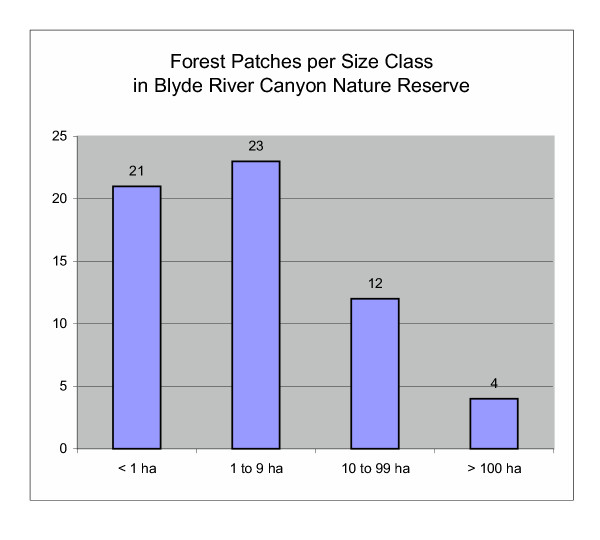
**Forest patches per size class. **Within the BRCNR, a total of 60 forest patches are recorded, varying from 0.21 ha to 567 ha in size.

We found a total area of 2111 ha of afromontane forest on BRCNR. This comprises 7.3% of the reserve's total surface area. Figure 2 shows the distribution of the forest on BRCNR. The forest is fragmented, with a total of 60 forest patches recorded, varying from 0.21 ha to 567 ha in size. The number of patches per size class illustrates this level of fragmentation (Figure 3). Unfortunately, 15 % of the larger forest patches have their patch size delimited by political boundaries. This means that a few forest patches continued over BRCNR's borders onto adjacent land and only the portions occurring on BRCNR were included in the calculations. If the average patch size is only calculated from patches with natural boundaries within the reserve, then the average size of forest patches is only 23 ha. This small patch size accentuates the fragmented nature of the afromontane forest on BRCNR.

### Flora and forest classification

**Figure 4:**
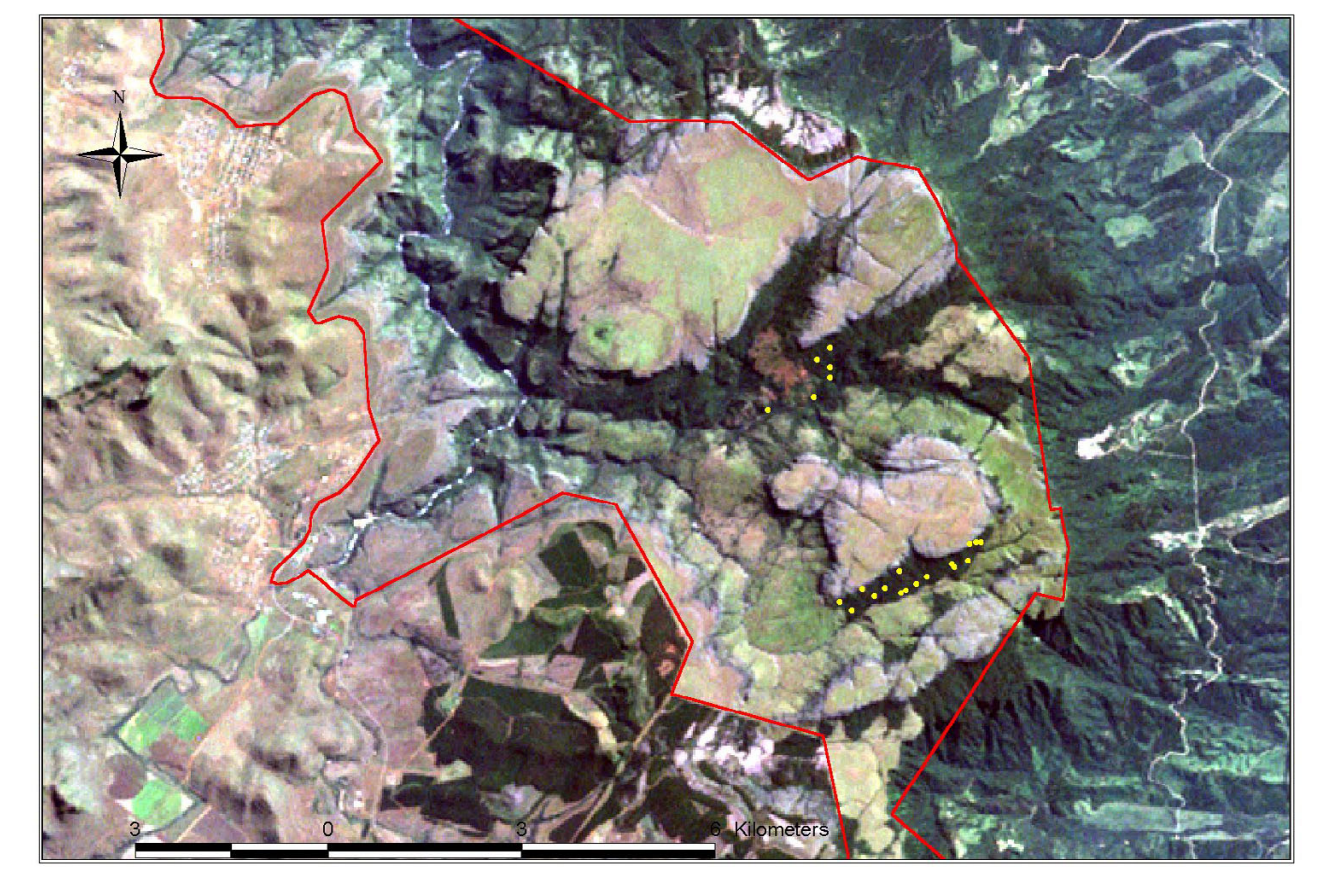
**Forest fragment study plots. **Distribution of forest study plots (yellow dots) within the Blyde River Canyon Nature Reserve boundaries (red lines) shown on Landsat 7 satellite image.

The forest flora recorded in 22 relevés is listed in Appendix A (see additional file 1). The total of 167 species includes 38 ferns and fern allies, 3 conifers and 140 flowering plants (11 monocots, 131 dicots). Previous botanical field work and logistical reconnaissance of the forest fragments within the park boundaries showed the forest flora to be qualitatively similar. However, some areas of the fragments were unreachable or technically difficult to visit and study due to the escarpment topography. For purposes of this vegetation analysis, the relevés were located (Figure 4) in two of the five largest forest patches, located in the central region of the BRCNR, where forest fragments were at least reasonably accessible. At this time, we consider these relevés to be representative. While the forest flora of BRCNR has not been exhaustively surveyed, this species list represents our best knowledge collected to date.

**Figure 5:**
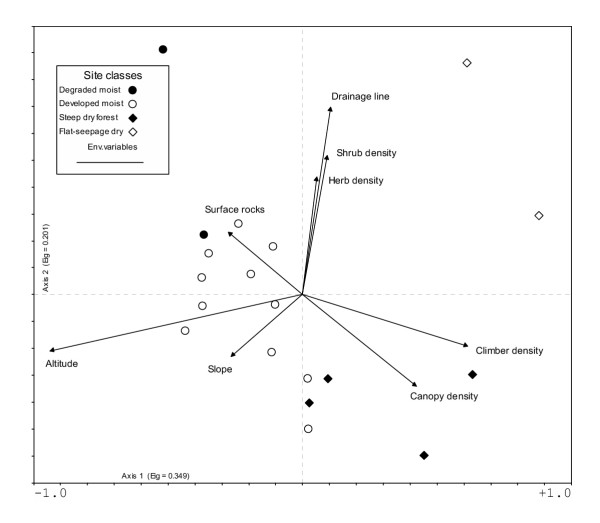
**Relationship between plant communities and environmental gradients. **A CANOCO joint plot showing the relationship between the proposed plant communities and environmental gradients. The length and position of the arrows indicate the strength of the relationship and the direction of change.

From our analysis of the forest vegetation in the relevés, two main plant communities – moist afromontane forest at high altitude and dry afromontane forest at lower altitude – are identified and described below. An eigenvalue of 0.33 was produced from the TWINSPAN algorithm at the first division into two communities. This value is strong enough to represent beta diversity and the division into two plant communities is accepted. Each community was then further broken down again into two communities, whose eigenvalues were lower and therefore taken as a measure of alpha diversity. The four proposed sub-communities are accepted as variants within the two larger communities. A complimentary analysis of forest communities using DECORANA was carried out and supports the results of the CANOCO analysis. Figure 5 displays the results from the CANOCO analysis. This joint plot data is analyzed for relationships with environmental data. These environmental variables are displayed as arrows radiating from the center of the diagram with the length of the arrows representing each environmental variable's contribution to explaining the variation in the sample scores on each axis. Altitude contributed most of the variation in samples along the bottom axis. Axis 1 is therefore based on an altitudinal gradient. Other environmental variables that contributed towards the floristic variation include drainage, slope, rockiness, shrub density, herb density, climber density and canopy density. The effects of aspect and topography were excluded, as their contribution was small. An altitudinal gradient accounts for most of the variation within the forest communities.

#### Moist high-altitude afromontane forest

This was a tall forest community that was heavily logged at the turn of the 20^th ^century. The canopy is still very uneven as the sub-canopy trees have not yet matured or replaced those trees which were originally removed. This forest occurs in the mist-belt along the escarpment at altitudes of between 1450 m and 1700 m. The slopes are steep and generally scattered with large boulders. Preferential species identified for this community include *Clivia caulescens*, *Ochna arborea*, *Podocarpus latifolius*, *Rapanea melanophloeos*, *Vernonia wollastonii *and *Blechnum attenuatum*. Epiphytes are common in these forests. Within this community, two variants are recognized based on canopy development.

#### Degraded moist high-altitude forest

A very broken and open canopy with a large shrub component characterizes this forest type. A number of species within this community are shade-intolerant and have established themselves under an open canopy. This sub-community has been so over-utilized that the canopy has never been able to close and this community is sometimes dominated by the exotic invader species *Acacia mearnsii*. Other preferential species include *Rubus *spp., *Myrsine africana *and *Helichrysum chrysargyrum*. This community is only temporary as it will eventually be replaced by climax species during the next seral stage when the canopy closes.

#### Developing moist high-altitude forest

This forest type has a closed canopy with only a few pioneer, shade-intolerant species, such as *Rhus tumulicola*, *Maesa lanceolata *and *Acacia mearnsii*, found in the canopy. The shrub component is not nearly as dense as the degraded sub-community community. Preferential species include *Behnia reticulata*, *Dovyalis lucida*, *Olea capensis *subsp. *macrocarpa*, *Asplenium rutifolium *and *Jasminum abyssinicum*.

#### Dry low-altitude afromontane forest

Parts of this forest type were also logged at the turn of the 20^th ^century. This dry forest community occurs just below the escarpment mist-belt, from 1200 m to 1450 m above sea level. Slopes are steep to gentle, with scattered large boulders. Preferential species in this forest community include *Brachylaena transvaalensis*, *Podocarpus falcatus*, *Adenopodia spicata*, *Lauridia tetragona *and *Pteris captoptera*. Within this community, two variants are recognized based on degree of slope.

#### Dry forest on gentle slopes or along drainage lines

This forest type occurs in areas with a gentle slope or along drainage lines. Both the shrub and herb layer densities are high with the increase in soil moisture gained directly from drainage lines or as a result of poor drainage on gentle slopes. Differential species include *Acacia ataxacantha*, *Combretum kraussii*, *Eugenia natalitia*, *Gymnosporia mossambicensis *and *Faurea galpinii*.

#### Dry forest along steep slopes

These low altitude forests occur along steep slopes. Overall canopy density is high with a poorly developed herb layer. This sub-community eventually grades into the moist high-altitude afromontane forest at higher altitudes. Preferential species include *Combretum edwardsii*, *Cryptocarya transvaalensis*, *Chionanthus peglerae*, *Xymalos monospora*, *Oxyanthus speciosus *and *Prosphytochloa prehensilis*.

### Species responses

**Figure 6:**
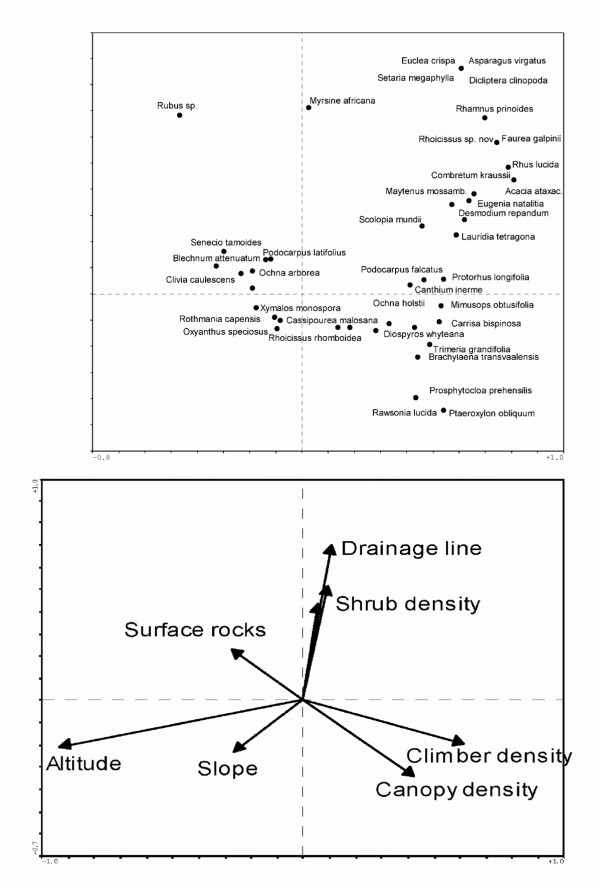
**Species responses to environmental variables. **Upper graph is a scatter diagram produced from CANOCO using the CCA procedure, displaying species responses to environmental variables. Lower graph displays strength and position of environmental variables.

Our analysis showed that altitude appears to be the primary environmental variable responsible for the distribution of forest plant species along environmental gradients. Specific species responses to environmental variables are depicted in Figure 6. With 167 species recorded in the plots, only those species with a cumulative fit of more than 0.35 are displayed. Canopy density is used as a surrogate for measuring disturbance, or forest dynamics. It is expected that forest dynamics would have an influence on the distribution of forest species.

*Asparagus virgatus*, *Dicliptera clinopodia*, *Setaria megaphylla and Desmodium repandum *are a few of the herbaceous plants that prefer the lower lying forests. Some of the trees include *Euclea crispa*, *Rhamnus prinoides*, *Combretum kraussii*, *Myrsine africana*, *Lauridia tetragona*, *Protorhus longifolia *and *Scolopia mundii*. Species that occur in the higher lying forests include *Oxyanthus speciosus*, *Xymalos monospora*, *Rothmannia capensis *and *Cassipourea malosana*. Some of the species that appear to prefer rocky areas include *Clivia caulescens *and *Blechnum attenuatum*. On the other hand, *Prosphytochloa prehensilis, Rawsonia lucida *and *Ptaeroxylon obliquum *seem to favor areas almost devoid of rocks or boulders. Species that favor low canopy densities, or higher levels of disturbance, are *Rubus *sp., *Blotiella natalensis*, *Acacia mearnsii *and *Schefflera umbellifera*.

## Discussion

Geldenhuys [1] states that species diversity within forest patches is determined by patch size and proximity to other forests, which together explain 82% of the species richness in South Africa's forests. The floristic variation within BRCNR's forests can be attributed predominantly to variation in species composition along an altitudinal gradient. Extremes in environmental variables and gradients, associated with a history of over-utilization, have resulted in a floristically and dynamically diverse forest type. Fire exclusion, clearing of old plantation sites, and a history of intensive and selective logging have all contributed to the variation in forest dynamics currently occurring on BRCNR.

The afromontane forests occurring on BRCNR are extremely fragmented, and yet over 2100 ha of forest patches are conserved within its boundaries. A large proportion of forest-dependent tree species are offered protection within BRCNR's borders. Of special interest was the discovery of *Jasminum abyssinicum*, *Combretum edwardsii *and *Olinia radiata*, all of which occurred in relatively large numbers in the forest. *Jasminum abyssinicum *had a status of Insufficiently Known under the old Transvaal threatened plants programme [15], and Hilton-Taylor [16] currently lists this species under the Red Data List of South African Plants. *Jasminum abyssinicum *occurred in 64% of the sample plots and in both forest communities identified. *Combretum edwardsii *is similarly listed as Insufficiently Known and also occurs under the Red Data List of South African Plants. *Combretum edwardsii *occurred in 73% of the sample plots. Pooley [17] lists *O. radiata *as very rare in KwaZulu Natal and Transkei and fails to mention its occurrence north of Natal. Palmer & Pitman [18] describe *O. radiata *as a rare species restricted to Natal forests from Pondoland to Zululand. This species was surprisingly common in the canopy of BRCNR's forests and occurred in 82% of the sample plots.

Some severely over-utilized forest patches are currently in a state of recovery; however, current and future damage from invasive trees is a threat to this recovery. Some of the forest patches on BRCNR have a forest margin largely comprised of the invasive black wattle (*Acacia mearnsii*). The flammable nature of this species, compared to natural forest margin species, allows grassland fires to penetrate forests, resulting in a reduction in forest patch size. With a large edge effect resulting from many small forest patches, there is a need for careful fire management and stringent alien plant control.

According to Geldenhuys [5], conservation status implies the extent to which populations, species or communities have been modified by the influences of man and the degree to which they might be expected to maintain their genetic diversity and ecological processes in the medium term (10 to 100 years). We see two different aspects to the conservation of the afromontane forest biome in the BRCNR. Firstly, it is the maintenance of the components and critical processes within a forest ecosystem. The disturbed and unstable state of the forest margins are identified as an area requiring further investigation. The effects of the alien tree species (e.g., black wattle) and the destructive burning of forest margins should be of concern to management authorities, as the forest patches are being reduced in size and the impact of edge effects is being amplified on the forest interior. Secondly, it is the maintenance of gene flow between the fragmented forest patches through management of the land surrounding the forest and forest corridors. As the BRCNR forest vegetation is situated along an altitudinal gradient, it therefore seems possible to identify certain forest patches (which may have been harvested in the past, or possibly will in the future) as critical adjuncts for conserved forest patches at the same altitude.

From the evidence we gathered, no "climax" forests exist on BRCNR. Although it is known that BRCNR's forests were utilized, the impact, extent and degree of the utilization are still not quantified. An investigation into the successional and dynamic state of the five largest forest patches is currently underway. Very little of the neighbouring forest on Mariepskop was harvested for its timber, and this forest seems to be the obvious control site for further comparative research.

## Conclusions

This study shows that BRCNR has a fragmented network of small forest patches that together make up 7.3% of the reserve's surface area, almost twice the area that was generally known. Two afromontane forest communities are recognized and associated along an altitudinal gradient, one within the moist mist belt and one within drier micro-climates outside the mist belt; further, within each community, variants were recognized based on either available soil moisture or degree of past utilization. These forest patches host a variety of forest-dependent trees, including some species considered rare, insufficiently known, or listed under the Red Data List of South African Plants. The fragmented nature of the relatively small forest patches accentuates the need for careful fire management and stringent alien plant control.

## Methods

### Forest size and fragmentation

All forest patches greater than 0.25 ha in size were mapped on a Geographical Information System (GIS). Forest boundaries were marked on 1:10 000 orthophotos, and the data were digitized onto the Mpumalanga Parks Board's GIS program, SPANS [19]. Total forest size and patch numbers are calculated from the digitized data.

### Floristic description

Twenty-two plots (0.04 ha each) were subjectively distributed throughout two of the five largest forest patches occurring on BRCNR (Figure 4), including the Op-de-Berg and Hebronberg forests. Plots covered an altitudinal range from 1240 to 1660 m above sea level and were sampled along environmental gradients, including the factors of slope, surface rockiness, drainage, topography, and disturbance (from logging). Sample relevés included a list of all the species present in a sample plot as well as cover-abundance values for each species, according to the Braun Blanquet cover-abundance scale [20]. A forest flora (see additional file 1 – Appendix A) is compiled from the species composition lists recorded in the 22 relevés.

### Data processing and analyses

Eigenvalues produced from the TWINSPAN and DECORANA programs are a measure of the degree of separation in the data. According to Jongman, ter Braak & van Tongeren [21], low eigenvalues would represent a poor separation of samples and can be regarded as a measure of alpha diversity (species turnover within a plant community). High eigenvalues would represent a strong separation of samples, which can therefore be a measure of beta diversity (species turnover between plant communities).

The 22 relevés were analyzed for a circumscription of possible plant communities. Firstly, a complementary analysis was run using the hierarchical classification program TWINSPAN [22,23] and the indirect ordination program DECORANA [24,25]. Complementary analyses of the two different multivariate analysis results provide for an accurate interpretation and description of plant communities [26]. Qualitative cover-abundance values were used for data input instead of quantitative values. Appendix A lists the 167 species recorded in the forest and used in the classification of plant communities. Secondly, the relevés were analyzed for relationships between plant communities and environmental gradients. Canonical correspondence analysis is a direct ordination technique that analyzes and presents such relationships between many species and numerous environmental variables. For this study, we used the program CANOCO [27]. No formal syntaxonomical classification was done in this study. An informal classification was performed with preferential species indicating the names of different vegetation associations.

Specific species responses to environmental variables are a useful way of displaying the impact a certain environmental variable may have on a species. Direct ordinations relate species presence to environmental variables on the basis of species and environmental data from the same set of sample plots [28]. A scatter diagram produced from the CANOCO program depicts the relationship between species and environmental variables. Only those species with a cumulative fit of more than 0.35 are displayed. This ensures that only those species that most positively contributed to the ordination scores in the scatter plot are displayed. Canopy density is used as a surrogate for measuring disturbance, or forest dynamics.

## Authors' contributions

ML conducted the field work and the map digitization. Both authors performed the statistical analyses. Both authors read and approved the final manuscript.

## Supplementary Material

Additional File 1 Appendix 1. Forest flora in 22 relevés on Blyde River Canyon Nature Reserve Click here for file
