# Supplementary material for: Preliminary inventory and classification of indigenous afromontane forests on the Blyde River Canyon Nature Reserve, Mpumalanga, South Africa
Source: BMC Ecol. 2004 Aug 2;4:9. doi: 10.1186/1472-6785-4-9 (PMC512296; doi:10.1186/1472-6785-4-9)
Supplement: Additional File 1 — Appendix 1. Forest flora in 22 relevés on Blyde River Canyon Nature Reserve [file 1472-6785-4-9-S1.pdf]

Appendix 1. Forest flora in 22 relevés on Blyde River Canyon Nature Reserve.
* = Exotic Species.

PTERIDOPHYTA
LYCOPSIDA
LYCOPODIACEAE
Lycopodium dacrydioides

SELAGINELLACEAE
Selaginella kraussiana

FILICOPSIDA
ASPLENIACEAE
Asplenium aethiopicum
A. anisophyllum
A. boltonii
A. erectum
A. friesiorum
A. lobatum
A. monanthes
A. rutifolium
A. sandersonii
A. splendens

BLECHNACEAE
Blechnum attenuatum
B. punctulatum

CYATHEACEAE
Cyathea capensis

DENNSTAEDTIACEAE
Blotiella natalensis

DRYOPTERIDACEAE
Athyrium scandicinum
Dryopteris inaequalis
D. squamiseta
Polystichum macleae
Rumohra adiantiformis

GLEICHENIACEAE
Gleichenia umbraculifera

HYMENOPHYLLACEAE
Hymenophyllum tunbrigense
Trichomanes borbonicum

LOMARIOPSIDACEAE
Elaphoglossum acrostichoides
E. aubertii
E. macropodium

MARRATIACEAE
Marattia fraxinea

OSMUNDACEAE
Osmunda regalis
Todea barbara

POLYPODIACEAE
Pleopeltis excavata
P. macrocarpa
P. schraderi
Polypodium polypodioides

PTERIDACEAE
Cheilanthes viridis var. *viridis*
Pteris catoptera

THELYPTERIDACEAE
Thelypteris bergiana

VITTARIACEAE
Vittaria isoetifolia

SPERMATOPHYTA
GYMNOSPERMAE
PINOPSIDA
PINACEAE
* *Pinus patula*

PODOCARPACEAE
Afrocarpus falcatus
Podocarpus latifolius

ANGIOSPERMAE
MONOCOTYLEDONAE (LILIOPSIDA)
POACEAE
Oplismenus hirtellus
Prosphytochloa prehensilis
Setaria megaphylla

CYPERACEAE
Cyperus albostriatus
C. leptocladus

AMARYLLIDACEAE
Clivia caulescens

IRIDACEAE
Dietes iridioides

ASPARAGACEAE
Asparagus setaceus
A. virgatus

LUZURIAGACEAE
Behnia reticulata

SMILACACEAE
Smilax anceps

DICOTYLEDONAE (MAGNOLIOPSIDA)
PIPERACEAE
Peperomia retusa
P. tetraphylla
Piper capense

ULMACEAE
Celtis africana

MORACEAE
Ficus craterostoma

PROTEACEAE
Faurea galpinii
F. macnaughtonii

SANTALACEAE
Osyridicarpos schimperianus

MENISPERMACEAE
Cissampelos torulosa

TRIMENIACEAE
Xymalos monospora

LAURACEAE
Cryptocarya transvaalensis
Ocotea bullata
O. kenyensis

BRASSICACEAE
Cardamine africana

ESCALLONIACEAE
Choristylis rhamnoides

PITTOSPORACEAE
Pittosporum viridiflorum

HAMAMELIDACEAE
Trichocladus grandiflorus

ROSACEAE
Rubus sp.

MIMOSACEAE
Acacia ataxacantha
* *A. mearnsii*
Adenopodia spicata

FABACEAE
Calpurnia aurea
Dalbergia armata
Desmodium repandum

RUTACEAE
Clausena anisata
Zanthoxylum davyi

PTAEROXYLACEAE
Ptaeroxylon obliquum

MELIACEAE
Ekebergia capensis

ANACARDIACEAE
Protorhus longifolia
Rhus chirindensis
R. tumulicola
R. lucida

AQUIFOLIACEAE
Ilex mitis

CELASTRACEAE
Elaeodendron croceum
Gymnosporia mossambicensis
Lauridia tetragona
Maytenus acuminata
M. peduncularis
M. undata
Pterocelastrus rostratus
Robsonodendron eucleiforme

ICACINACEAE
Apodytes dimidiata

SAPINDACEAE
Hippobromus pauciflorus

MELIANTHACEAE
Bersama tysoniana

BALSAMINACEAE
Impatiens hochstetteri

RHAMNACEAE
Rhamnus prinoides
Scutia myrtina

VITACEAE
Rhoicissus rhomboidea
R. sp.

TILIACEAE
Grewia occidentalis

OCHNACEAE
Ochna arborea var. *oconnorii*
O. holstii

FLACOURTIACEAE
Dovyalis lucida
Kiggelaria africana
Rawsonia lucida
Scolopia mundii
S. zeyheri
Trimeria grandifolia

BEGONIACEAE
Begonia sonderiana

OLINIACEAE
Olinia emarginata
O. radiata

THYMELACEAE
Peddiea africana

RHIZOPHORACEAE
Cassipourea malosana

COMBRETACEAE
Combretum edwardsii
C. kraussii

MYRTACEAE
Eugenia natalitia
E. woodii
Syzygium gerrardii

ARALIACEAE
Cussonia spicata
Schefflera umbellifera

CORNACEAE
Curtisia dentata

ERICACEAE
Vaccinium exul

MYRSINACEAE
Maesa lanceolata
Myrsine africana
Rapanea melanophloeos

SAPOTACEAE
Englerophytum magalismontanum
Mimusops obovata

EBENACEAE
Euclea crispa
Diospyros whyteana

OLEACEAE
Chionanthus foveolatus subsp. *major*
C. peglerae
Jasminum abyssinicum
Olea capensis subsp. *macrocarpa*
Schrebera alata

LOGANIACEAE
Nuxia congesta
N. floribunda
Strychnos henningsii

APOCYNACEAE
Carissa bispinosa

PERIPLOCACEAE
Secamone alpini

LAMIACEAE
Plectranthus fruticosus
P. laxiflorus
Stachys reticulata

SCROPHULARIACEAE
Bowkeria cymosa
Halleria lucida

GESNERIACEAE
Streptocarpus wilmsii

ACANTHACEAE
Dicliptera clinopodia
Mackaya bella
Sclerochiton harveyanus

Appendix 1. Forest flora in 22 relevés on Blyde River Canyon Nature Reserve.
* = Exotic Species.

RUBIACEAE
Canthium ciliatum
C. inerme
C. kuntzeanum
Cephalanthus natalensis
Galopina circaeoides
Oxyanthus speciosus
Pavetta inandensis
Psychotria capensis
P. zombamontana
Psydrax obovata
Rothmannia capensis
Tricalysia capensis

ASTERACEAE
Brachylaena transvaalensis
Conyza sp.
Helichrysum chrysargyrum
Senecio tamoides
Vernonia wollas
